# Supplementary figures and images for: Submembrane liprin-α1 clusters spatially localize insulin granule fusion
Source: J Cell Biol. 2025 Aug 28;224(10):e202410210. doi: 10.1083/jcb.202410210 (PMC12393827; doi:10.1083/jcb.202410210)

Fig 1H Source Data

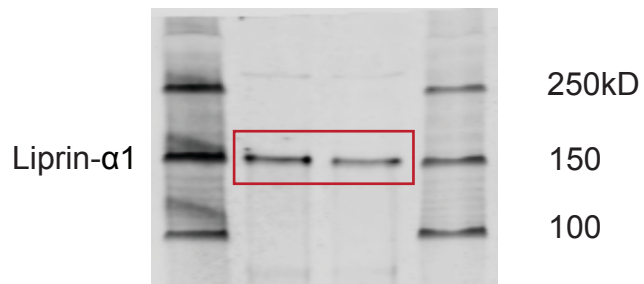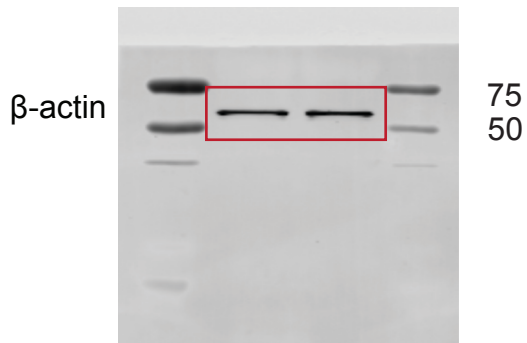

Supplement: SourceData F1 — is the source file for Fig. 1. [file jcb_202410210_sourcedataf1.pdf]

Fig 3B Source Data

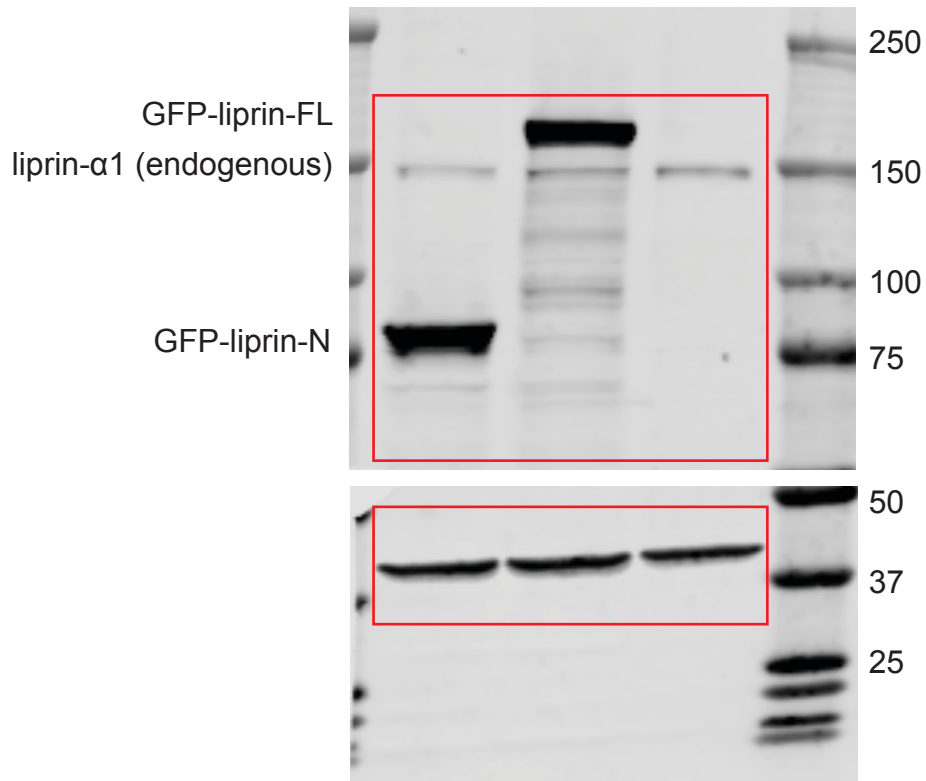

Supplement: SourceData F3 — is the source file for Fig. 3. [file jcb_202410210_sourcedataf3.pdf]

Fig 6C

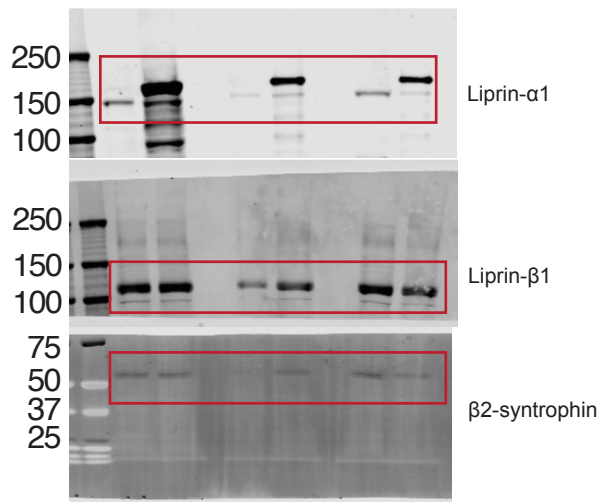

Fig 6D

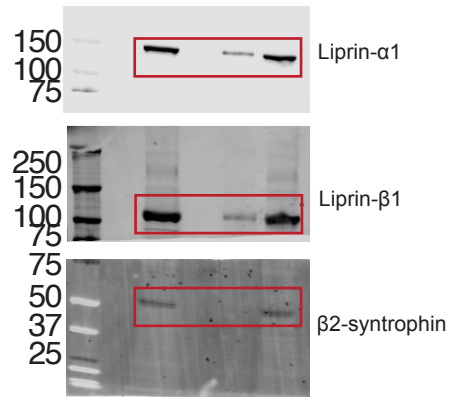

Supplement: SourceData F6 — is the source file for Fig. 6. [file jcb_202410210_sourcedataf6.pdf]
